# Plausible Mechanisms of Causation of Immediate Stroke by Cervical Spine Manipulation: A Narrative Review

**DOI:** 10.7759/cureus.56565

**Published:** 2024-03-20

**Authors:** Steven P Brown

**Affiliations:** 1 Integrative/Complementary Medicine, Brown Chiropractic & Acupuncture, PC, Gilbert, USA

**Keywords:** liability, malpractice, spontaneous internal carotid artery dissection, spontaneous vertebral artery dissection, causation, medicolegal, cervical spine manipulation, dissection, stroke, chiropractic

## Abstract

It has been proposed that cervical spine manipulation (CSM) can cause dissection in healthy cervical arteries, with resultant immediate stroke. However, research does not support a causal association between CSM and cervical artery dissection (CAD) in healthy cervical arteries. The objective of this study was to review the literature to identify plausible mechanisms of causation of immediate stroke by CSM. Immediate stroke is defined as a stroke occurring within seconds or minutes of CSM. Our review found plausible thromboembolic and thrombotic mechanisms of causation of immediate stroke by CSM in the literature. The common premise of these mechanisms is CAD being present before CSM, not occurring as a result of CSM. These mechanisms of causation have clinical and medicolegal implications for physicians performing CSM.

## Introduction and background

Cervical spine manipulation (CSM) is a manual therapy in which a controlled, high-velocity, low-amplitude thrust is applied to the cervical spine and induces a therapeutic stretch on the cervical spine joints. The majority of CSM performed in North America is done by chiropractic physicians; however, CSM is also performed by osteopathic physicians, naturopathic physicians, medical physicians, and doctors of physical therapy [1].

**Figure 1:**
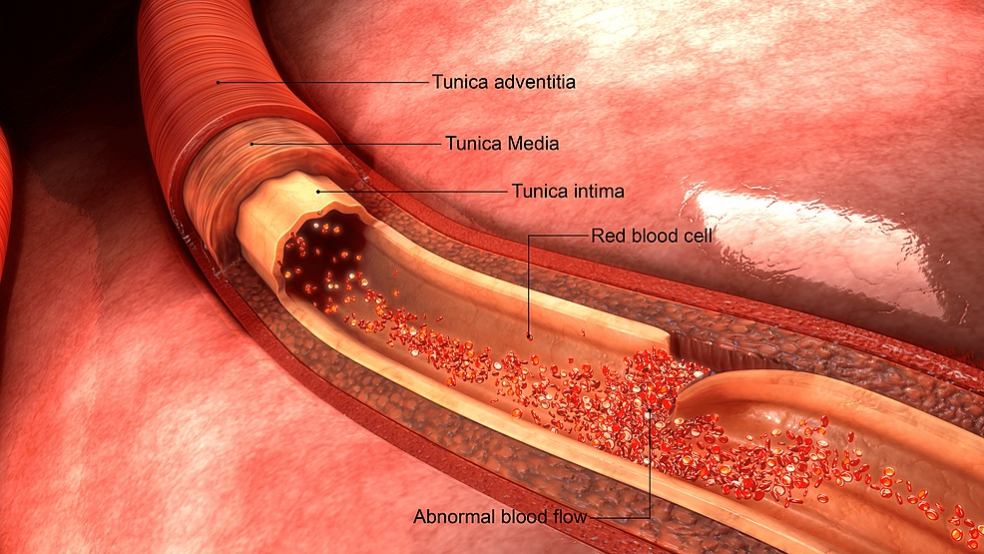
Anatomy of the cervical artery with intimal dissection. Image credit: sciencepics/Shutterstock.com.

The mechanisms of causation of stroke from CSM discussed in this paper involve cervical artery dissection (CAD). The cervical arteries are defined as the vertebral artery and the internal carotid artery. Anatomically, the arteries consist of three layers, namely, the tunica intima (inner layer), the tunica media (middle layer), and the tunica adventitia (outer layer). Arterial dissection refers to a flap-like tear in the inner lining of an artery, the tunica intima (Figure 1). The area of the intimal tear heals by forming a thrombus and may cause abnormal blood flow. Dissections may be traumatic or spontaneous. Traumatic dissections can result from severe trauma such as a motor vehicle collision or sports injury. Spontaneous dissections can occur with minor neck movements in the absence of any severe trauma. Spontaneous dissections are likely the result of an arteriopathy from an underlying connective tissue disorder causing arterial wall weakness.

The prevailing theorized mechanism of causation of stroke from CSM [2] is that the sudden movement and stretching of the cervical artery associated with CSM can cause CAD in an otherwise healthy cervical artery, resulting in immediate stroke. There are numerous case reports of stroke occurring immediately after CSM [3]. However, research does not support a causal association between CSM and CAD in a healthy cervical artery.

In 2008, Cassidy et al. found an increased risk of vertebrobasilar artery (VBA) stroke associated with doctor of chiropractic (DC) and primary care physician (PCP) visits and concluded that the increased risks of VBA stroke associated with DC and PCP visits are likely due to patients with headache and neck pain from VBA dissection seeking care before their stroke [2]. In 2017, Cassidy et al. reported similar findings regarding carotid artery stroke [4]. The findings of Cassidy et al. support that CSM does not cause CAD in cases of post-manipulative stroke and that CAD is likely pre-existing to the CSM.

In 2013, Symons and Herzog summarized the results of four biomechanical cadaver studies demonstrating that strains to the cervical arteries during CSM are typically less than 50% of strains obtained during normal range of motion (ROM) testing, and far less than failure strains [5]. In 2014, Biller et al. performed a literature review and concluded that biomechanical evidence was insufficient to establish the claim that CSM causes CAD [1]. Church et al. (2016) performed a systematic review and meta-analysis of published data on CSM and CAD. They concluded that there was no convincing evidence to support a causal link between CSM and CAD [6].

The most widely quoted estimate of the incidence of stroke and/or vertebral artery dissection after CSM comes from Haldeman et al. [7]. Haldeman et al. analyzed the incidence of stroke or vertebral artery dissection malpractice claims against DCs. They estimated an incidence of 1 in 5.85 million cervical manipulations. This figure is often referenced to show the rarity of post-manipulative stroke. However, as 1 million people visit DCs every day in the United States [8], this would indicate an incidence of every 5.85 business days in the United States. Not all chiropractic patients receive CSM, but even using a conservative estimate of 50%, this would indicate an incidence of every 11.7 days in the United States.

Chu et al. identified patients with spinal manipulative therapy (SMT)-related adverse events (AEs) from January 2017 through August 2022, a period of over five years [9]. They found 39 AEs and no cases of stroke or vertebral or carotid artery dissection during this 5+ year period. They concluded that severe SMT-related AEs were very rare. However, over the course of the 5+ years of this study, there were only 960,140 SMT sessions. Referencing the incidence ratio of Haldeman et al. of 1:5.85 million CSMs, the number of SMT sessions in the study by Chu et al. was insufficient to capture cases of dissection and/or stroke following CSM.

As it is not plausible that CSM could precipitate immediate post-manipulative stroke by causing dissection in an otherwise healthy cervical artery, this study aimed to review the literature to identify plausible mechanisms of causation of immediate stroke by CSM.

## Review

Methodology

English-language literature from the date of inception through March 2024 was searched from the following databases: PubMed and the Index to Chiropractic Literature. Search terms included chiropractic, stroke, dissection, “cervical spine manipulation,” “vertebral artery,” and “internal carotid artery.” Peer-reviewed or academic studies were included if the researchers proposed a plausible mechanism of causation of immediate stroke by CSM. Immediate stroke is defined as a stroke that occurs within seconds or minutes of CSM. Human and animal studies were considered.

Many well-known studies related to this topic were excluded. Rothwell et al. (2001) [10], Kosloff et al. (2015) [11], Church et al. (2016) [6], Cassidy et al. (2017) [4], Kennell et al. (2017) [12], Whedon et al. (2022) [13], and Whedon et al. (2023) [14] were excluded, as they did not propose any mechanism of causation of immediate stroke from CSM. Rothwell et al. did reference Norris et al. (2000) [15], which proposed a plausible mechanism of causation and was included in our study. Studies proposing that CSM may cause cervical artery stroke by way of vasospasm, hemostasis, subclinical endothelial injury, or turbulent flow [16] were excluded, as these mechanisms of causation have not been found plausible [17,18].

The potential for bias in this study was minimized by including an analysis of studies challenging the proposed mechanisms of a causal relationship between CSM and immediate post-manipulative stroke.

Results

A total of 12 studies met the search criteria, ranging in publication from 1989 to 2016 (Table 1). The lead researchers were seven neurologists, four chiropractors, and one physiotherapist. One neurologist, Haldeman, was also a chiropractor. Two plausible mechanisms of causation were found, thromboembolic and thrombotic.

**Table 1:** Studies proposing mechanisms of causation of stroke by cervical spine manipulation.

Year	Author(s)	Field	Design	Publication	Mechanism(s)
1989	Mas et al. [19]	Neurology	Case report	Neurology	Thromboembolic, thrombotic
1999	Haldeman et al. [20]	Neurology (Chiropractic)	Case series	Spine	Thromboembolic, thrombotic
2000	Norris et al. [15]	Neurology	Case series	Canadian Medical Association Journal	Thromboembolic
2002	Haldeman et al. [21]	Neurology (Chiropractic)	Case series	Journal of Neurology	Thromboembolic, thrombotic
2003	Smith et al. [22]	Neurology	Case control	Neurology	Thromboembolic, thrombotic
2008	Cassidy et al. [2]	Chiropractic	Case control	Spine	Thromboembolic
2009	Schwartz et al. [23]	Neurology	Case series	Journal of Stroke & Cerebrovascular Diseases	Thromboembolic, thrombotic
2011	Albuquerque et al. [24]	Neurology	Case series	Journal of Neurosurgery	Thromboembolic, thrombotic
2013	Tuchin [25]	Chiropractic	Review	International Journal of Clinical Practice	Thromboembolic
2015	Whedon et al. [26]	Chiropractic	Case cohort	Journal of Manipulative & Physiological Therapeutics	Thromboembolic
2016	Thomas [27]	Physical Therapy	Review	Manual Therapy	Thromboembolic, thrombotic
2016	Neeb and Reuter [28]	Neurology	Review	Treatment-Related Stroke	Thromboembolic, thrombotic

Mas et al. (1989)

In the earliest study found, Mas et al. [19] proposed that CSM could exacerbate CAD and result in stroke by thromboembolic or thrombotic mechanisms: “Thus, cervical pain that precedes and motivates chiropractic cervical manipulation may be the first symptom of a hitherto unrecognized spontaneous (or traumatic) dissection. In such a case, cervical manipulation would precipitate stroke by either worsening arterial damage, leading to positional occlusion of an already narrowed artery, or dislodging an intraluminal thrombus” (Table 2). This case reported by Mas et al. resulted in death. Mas et al. were able to determine from the autopsy that the dissection was present before the CSM and that the dissection was worse after CSM. They also determined that the most likely mechanism of stroke was thromboembolic.

**Table 2:** Mechanisms of causation of immediate stroke by cervical spine manipulation. CSM = cervical spine manipulation

Mechanism of causation	Description
Thromboembolic	Sudden neck movement from CSM could dislodge a loosely adherent cervical artery thrombus. The dislodged embolus may travel and occlude a smaller artery that supplies the brain
Thrombotic	An already large thrombus could be suddenly repositioned in such a way that it blocks the cervical artery, resulting in ischemic stroke from vascular occlusion

These were the only two plausible mechanisms of causation of stroke by CSM found in our literature search. Both the thromboembolic and thrombotic mechanisms require CAD to be present before CSM, not as the result of CSM. The remaining 11 studies published after Mas et al. support these mechanisms. As it has been denied that the research supports mechanisms of causation of stroke from CSM [29], direct quotes from these peer-reviewed studies are provided.

*Haldeman* *et al. (1999)*

Haldeman et al. [20] proposed thromboembolic and thrombotic mechanisms of stroke from CSM: “Because most cervical manipulations are administered to treat neck pain and headaches, these patients with a dissection in progress on seeing a practitioner are likely to be manipulated, and that in turn could precipitate a vascular occlusion or dislodge an embolus.”

Norris et al. (2000)

Norris et al. [15] proposed a thromboembolic mechanism of stroke from the sudden neck movement associated with CSM. Norris et al. stated that neck pain is a common symptom of the onset of CADs and observed: “These otherwise asymptomatic lesions will heal when left alone, but if there is vigorous neck movement or manipulation at a later date, a loosely adherent clot may dislodge and embolize to the brain.”

Haldeman et al. (2002)

Haldeman et al. [21] again proposed thrombotic and thromboembolic mechanisms of stroke from CSM: “It [our data] does, however, suggest that many of these dissections may be spontaneous or due to trivial trauma and that manipulation may be simply the final insult that precipitated the vascular occlusion or release of a thrombotic embolism.”

*Smith* *et al. (2003)*

Smith et al. [22] reasoned that if CSM was prompted by pain from CAD, it could contribute to the risk of stroke by either extending the dissection or dislodging an embolus. Smith et al. concluded: “From this case report and our study, it appears that SMT [spinal manipulative therapy] may exacerbate pre-existing dissections, producing immediate or delayed embolization.” Smith referenced a 1993 case report in which an autopsy demonstrated that in a case of post-manipulative stroke, the patient had vertebral artery dissection (VAD) before the CSM [30].

*Cassidy* *et al. (2008)*

In this study on VBA stroke, Cassidy et al. [2] proposed a thromboembolic mechanism of causation of stroke by CSM: “It might also be possible that chiropractic manipulation, or even simple range-of-motion examination by any practitioner, could result in a thromboembolic event in a patient with a pre-existing vertebral artery dissection.”

Cassidy et al. designed the study taking into account that CSM can cause immediate stroke: “For the chiropractic analysis, the index date was included in the hazard period, since chiropractic treatment might cause immediate stroke and patients would not normally consult a chiropractor after having a stroke” [2]. This same study design was used in the 2017 study by Cassidy et al. on carotid artery stroke [4].

*Schwartz* *et al. (2009)*

Schwartz et al. [23] supported thrombotic and thromboembolic mechanisms: “Certainly, patients may have visited a chiropractor for relief of neck pain that could have been secondary to a dissection, but even if this were the case, we cannot rule out that the adjustment acted as a ‘second hit,’ leading to worsening of the dissection or embolism from a stable dissection.”

*Albuquerque* *et al. (2011)*

Albuquerque et al. [24] reasoned that: “Patients often visit the chiropractor complaining of head or neck pain, and a certain percentage may have preexisting arterial dissections.” “Nonetheless, as demonstrated in this series, patients can present within hours to days of chiropractic manipulation with new objective neurological deficits or more severe neurological complaints. This temporal relationship suggests that either the arterial injury was produced de novo or made worse as a result of manipulation.” As there is no convincing evidence that CSM can produce arterial injury de novo in a healthy artery [6], it follows from Albuquerque et al.’s argument that an existing dissection was made worse as a result of manipulation. Albuquerque et al. proposed thromboembolic and thrombotic mechanisms: “Stroke is produced either by propagation of a thrombus from the dissected arterial segment or by severe dissection-induced arterial stenosis and secondary ischemia.”

Tuchin (2013)

Tuchin [25] proposed a plausible thromboembolic mechanism of causation: “Physical triggers, including SMT, can serve as plausible final link between the underlying disease and stroke (for instance, in case of arterial dissection with existing connective tissue weakness).”

Tuchin observed that coherence is necessary when formulating a mechanism of causation [31]. Coherence describes the need for any causal association to be compatible with the existing theory and knowledge. In the past, it was accepted that CSM could cause CAD in a healthy cervical artery and precipitate immediate ischemic stroke; this mechanism of causation did not contradict an accepted theory [15]. After recent studies showing no convincing evidence of a causal association between CSM and CAD in a healthy cervical artery, an alternative mechanism of causation is needed to explain the phenomenon of immediate post-manipulative stroke.

A clinical setting could arise in which a patient develops CAD from factors such as minor trauma, sports, sustained neck positions, or excessive movements. This patient could have been predisposed to CAD by an arteriopathy (possibly transient) due to hypertension, hyperlipidemia, hyperhomocysteinemia, recent infection, smoking, diabetes, migraine, or other combinations of factors. The patient could experience neck pain and/or headache from CAD and seek manipulative treatment from a DC, physiotherapist, or some other healthcare practitioner. Tuchin concludes: “If this healthcare practitioner did not take a thorough clinical history, then they may overlook these above factors and perform an SMT when it may have been contra-indicated. Therefore, an existing VAD is exacerbated, the thrombus is dislodged and creates the stroke” [25].

*Whedon* *et al. (2015)*

Whedon et al. concluded that CSM is unlikely to be a significant cause of stroke in older adults [26]. In arriving at this conclusion, Whedon et al. took into account a plausible thromboembolic mechanism of causation of stroke by CSM: “Because vertebral artery dissection and associated thromboembolism are the most plausible mechanism by which spinal manipulation could cause stroke, our findings support current best evidence suggesting that manipulation of the cervical spine is unlikely to be a significant cause of stroke in older adults.”

Thomas (2016)

Thomas [27] proposed a thromboembolic mechanism of immediate post-manipulative stroke: “Manipulative therapy performed when a dissection is present could further damage the artery or propagate an embolus.” Thomas specified that this mechanism of causation is only plausible with a close temporal relationship between CSM and the onset of ischemic stroke symptoms.

Neeb and Reuter (2016)

Neeb and Reuter observed that CAD that leads to stroke in a temporal relationship to CSM is not necessarily caused by CSM. Patients with spontaneous CAD commonly experience neck pain which is often misdiagnosed as pain of musculoskeletal origin. For the treatment of neck pain, these patients may seek CSM which could “…contribute to the risk of stroke by exacerbating the pre-existing dissection or dislodging an embolus” [28].

Wynd et al. (2008): Contrary Study

One contrary study was found. The results of this study disputed the proposition that CSM could affect an existing CAD. Wynd et al. found that CSM did not alter the dimensions of pre-existing vertebral artery lesions in anesthetized dogs [32]. However, this canine study had substantial limitations.

Wynd et al. investigated vertebral artery lesions at the C4 level, not the C0-C2 level, the site of the commonly affected V3 segment in humans. Moreover, the time from the creation of the cervical artery lesion to the CSM was not documented but appears to be short. Many strokes following CSM occur weeks after the onset of symptoms of CAD, likely because a healing thrombus becomes more loosely adherent and easier to dislodge and embolize to the brain [15].

Allegedly contrary studies

Cassidy et al. (2008) [2], Cassidy et al. (2017) [4], Kosloff et al. (2015) [11], and Whedon et al. (2015) [26] have been referenced to support no causal relationship between CSM and post-manipulative stroke [25]. However, the conclusions of these studies do not apply to immediate post-manipulative stroke.

A limitation of these epidemiological studies based on administrative insurance claims data is establishing temporality. Such claims data can establish whether stroke and CSM occurred on the same day, but cannot establish the time lapse from CSM to stroke, and cannot establish whether the stroke occurred before or after CSM. Therefore, these studies cannot establish temporality to construct an immediate cohort. A 0-1-day cohort is normally the shortest cohort possible. However, researchers must *speculate* what came first, the CSM or the stroke. Therefore, the conclusions of these studies as regards the 0-1-day cohort have substantial limitations. Cassidy et al. [2] and Kosloff et al. [11] did not rule out a causal association between CSM and stroke, and Whedon et al. [26] noted that a thromboembolic mechanism of causation was plausible.

Cervical spine manipulation and the susceptible cervical artery

While it is not plausible that CSM could cause CAD in a healthy cervical artery, it is plausible that CSM could cause CAD in a susceptible cervical artery [25]. As noted by Tuchin, a susceptible cervical artery would be one predisposed to dissection by an arteriopathy [25]. It has been proposed that the use of fluoroquinolones to treat acute respiratory infections may make cervical arteries susceptible to dissection [33].

If CSM did cause CAD in such a susceptible patient, it would not be likely to cause immediate stroke by a thromboembolic or thrombotic mechanism. It is not plausible for a thrombus to instantly form, dislodge, travel to the brain, and cause a stroke within seconds or minutes of CSM [25]. Normal clotting time is 4-10 minutes [34]. It would then take more time for a thrombus to accumulate, dislodge, and embolize to occlude a smaller artery that supplies the brain, resulting in a thromboembolic stroke. Moreover, the formation of large thrombi that can occlude an artery, resulting in a thrombotic stroke, also requires time.

The immediate consequence of CAD would be sudden neck pain and/or headache, a brief syncope, and perhaps nausea, vertigo, and tinnitus [35]. A stroke is not likely to occur immediately, and if it occurs at all, it would not be until hours or days later due to enlargement of the dissection or propagation of a thrombus.

Clinical implications

Plausible mechanisms of causation of stroke from CSM have clinical implications for physicians performing CSM in three primary areas: i.e., informed consent, history and examination, and diagnosis. First, as there is a risk of stroke from CSM, and the potential consequences are severe [36], verbal and written informed consent to the risk of thromboembolic or thrombotic stroke from CSM should be obtained [37].

Second, a thorough history taking and examination should be performed and documented. History taking, especially regarding the time of symptom onset, is the single most important factor for detecting CAD [38]. There is no objective screening test for CAD [39]. The physician must have clinical knowledge of the subjective symptoms of CAD [40], as well as a high index of clinical suspicion [41].

Third, there should be a diagnostic assessment to exclude CAD before performing CSM [38]. The physician should consider CAD whenever neck pain, headache, or dizziness are present, even if they are the only presenting symptoms [40]. When neck pain and headache are characteristic of CAD, there should be an immediate referral to the medical emergency department. Characteristics of neck pain and headache resulting from CAD are new, ipsilateral sub-occipital neck pain and distinct, new, continued headache [38]. Continued headache deserves special clinical consideration. Episodic stress/tension headaches [42] and migraines [43] normally resolve or improve after 72 hours or less. Headache continued or worsening after 72 hours should be considered a potential symptom of CAD [44].

Implications for physician liability

In cases of immediate thromboembolic or thrombotic post-manipulative stroke, there are three primary clinical settings to consider when evaluating the liability of the physician for the causation of the stroke.

Clinical Setting 1

Patients presenting with existing CAD with symptoms of neck pain and/or headache accompanied by neurological signs and symptoms: The CSM provider would likely be liable for causation of the stroke if they did not document a thorough history and examination that excluded CAD [38].

Neurological signs and symptoms are red flags that the patient’s condition may not be musculoskeletal. The physician should determine the nature of the neurological signs and symptoms before proceeding with CSM. When neck pain and/or headache are characteristic of CAD and ischemic symptoms are present, there should be an immediate referral to the medical emergency department [38]. The physician should not perform CSM. If a patient is having ischemic symptoms as a result of CAD, then, by definition, they are having an ischemic stroke as a result of CAD [45].

Clinical Setting 2

Patients presenting with existing CAD with symptoms of neck pain and/or headache: The CSM provider would likely be liable for causation of the stroke if they did not document a thorough history and examination that excluded CAD.

CAD should be considered in a diagnostic assessment whenever neck pain and/or headache are present, even if they are the only presenting symptoms [46]. When neck pain and/or headache are characteristic of CAD, there should be an immediate referral to the medical emergency department [38]. The physician should not perform CSM.

Clinical Setting 3

Patients presenting with existing CAD with no signs or symptoms [29]: Assuming the CSM provider performed and documented a thorough history and physical examination, the CSM provider would likely not be liable for causation of an immediate thromboembolic or thrombotic stroke. A physician is not likely to be held liable for aggravating CAD they cannot suspect or diagnose.

However, a case report of immediate post-manipulative stroke in a patient verified to have no prior neck pain, headache, or neurological symptoms was not found. Case reports alleging no prior neck pain, headache, or neurological symptoms should be reviewed carefully. Errors and omissions have been noted in case reports of adverse effects of spinal manipulation [47]. Notably, the symptoms prompting the patient to seek CSM are not documented because medical records before the stroke were not reviewed.

A patient may present with an asymptomatic CAD which was formerly symptomatic. The healing arterial wall may become asymptomatic with the thrombus still present. The physician may fail to diagnose the currently asymptomatic CAD if they do not obtain a thorough history to discover a recent history of neck pain and/or headache characteristic of CAD. Lee et al. reported three cases of asymptomatic CAD [48]. However, medical records were not reviewed for a recent history of neck pain and/or headache characteristic of CAD.

It seems plausible that CAD could be so minor that it never generates symptoms and heals on its own. However, it does not seem likely that such a minor CAD could generate a thrombus significant enough to cause thromboembolic or thrombotic stroke. No research into this possible clinical setting was found, likely because an asymptomatic condition is difficult to detect.

Haldeman et al. (2002)

In their 2002 case series, Haldeman et al. were unable to identify factors from the clinical history and physical examination of the patient that would assist a physician attempting to isolate the patient at risk of stroke after CSM. Haldeman et al. concluded that strokes after CSM are unpredictable and should be considered an inherent, idiosyncratic, and rare complication of CSM [49].

If there are no clinical factors that can assist the physician in identifying patients at risk of stroke after CSM, then CSM should not be performed. The potential risks, i.e., paralysis and death, would outweigh any benefits. However, patients at risk of stroke after CSM are those who have pre-existing CAD. Clinical strategies to exclude CAD before performing CSM have been published by the medical [38], chiropractic [38], and physical therapy [50] professions and can assist the physician in identifying patients at risk of stroke after CSM.

Implications for medicolegal causation

Although plausible mechanisms of causation of immediate stroke by CSM have been reported in the literature, advanced research on these mechanisms to determine causation, such as randomized controlled trials (RCTs), has not been done. RCTs would be infeasible and unethical in these clinical settings due to the rarity of CAD and the life-threatening nature of stroke [38]. As RCTs are infeasible, the next best external evidence that is available must be used [51].

In the absence of RCTs to determine causation, medicolegal causation can be established to a standard of more likely than not if plausibility, temporality, and lack of a more probable alternative explanation are present [52]. The three criteria presented in Table 3 should be met to establish causation of stroke by CSM as more likely than not.

**Table 3:** Criteria for medicolegal causation of stroke by cervical spine manipulation. CAD = cervical artery dissection; CSM = cervical spine manipulation

Criteria	Description
1. Plausibility	There should be pre-existing CAD. Thromboembolic and thrombotic mechanisms of causation of immediate stroke by CSM are plausible in the presence of CAD
2. Temporality	There should be a close temporal association (seconds or minutes) between CSM and the onset of ischemic stroke symptoms
3. Lack of a more probable explanation	There should not be a more probable alternative explanation for the cause of the post-manipulative stroke. If it is hours, days, or weeks after CSM before the onset of ischemic stroke symptoms, there could be a more probable alternative explanation for the cause of the stroke

A confounder is any unmeasured variable that may make a purported causative relationship correlative or associative [6]. If a confounder is present, there may be a more probable alternative explanation for the alleged causal relationship. However, in the case of an immediate thromboembolic or thrombotic stroke after CSM performed in the presence of CAD, the presence of a confounder is unlikely. In cases of non-immediate stroke after CSM, the presence of a confounder is more likely.

Alternative Explanation 1

One alternative explanation is that it is a coincidence that the stroke occurred after CSM. It was a spontaneous event unrelated to the CSM. However, this is not a more probable alternative explanation for an immediate post-manipulative stroke.

In many cases, CAD is present and stable for days or weeks before stroke occurs. It is only immediately after CSM that ischemic symptoms of stroke begin. It is highly improbable that a patient will have a stroke and have had CSM within seconds or minutes purely by chance given the relatively low frequency of both events [22]. The most probable explanation is that CSM caused the stroke by a thromboembolic or thrombotic mechanism.

Protopathic bias has been proposed to explain the association between CSM and stroke [4]. Protopathic bias occurs when an exposure (CSM) is delivered in the presence of one disease (CAD causing neck pain and/or headache) before a second disease occurs (thromboembolic of thrombotic ischemic stroke from CAD). In case-control studies, protopathic bias can lead to the conclusion that the exposure (CSM) caused the outcome (stroke), even though CSM was not on the causal pathway.

In cases of non-immediate stroke, where stroke follows CSM within hours, days, or weeks, protopathic bias can explain the association between CSM and stroke. This is because there is no causal pathway for a non-immediate stroke following CSM, there is no temporality, and there could be a more probable alternative explanation for the cause of a non-immediate stroke.

However, in cases of immediate stroke following CSM, there are plausible thromboembolic and thrombotic causal pathways, there is temporality, and there is not likely to be a more probable alternative explanation. There is a basis for medicolegal causation in the case of an immediate stroke.

Alternative Explanation 2

When ischemic symptoms begin immediately after CSM, but after further neck movements have occurred, another alternative explanation is that the further neck movements dislodged or repositioned a loosely adherent cervical artery thrombus. This may or may not be a more probable alternative explanation, depending on the type of further neck movements.

If neck movements after CSM exert more strain on a cervical artery than CSM, then the neck movements may be a more probable alternative explanation. Research supports that movements involving the full possible ROM exert more strain on a cervical artery than CSM [5]. If it can be shown that the patient performed neck movements involving full possible cervical spine ROM, then those neck movements may be a more probable alternative explanation.

A 2023 study concluded that CSM exerts no stretch on the vertebral artery, it simply elongates it [53]. In this case, it may be that any neck movement that elongates the vertebral artery more than CSM is a more probable alternative explanation. Movements involving the full possible cervical spine ROM would elongate the vertebral artery more than CSM.

Alternative Explanation 3

Another alternative explanation that has been offered is that stroke immediately following CSM is not causally related to CSM because stroke is the inevitable result of CAD regardless of CSM. Murphy opined that there was an inevitable progression from dissection to stroke and this natural progression from dissection to stroke appears to occur independent of the application of CMT [29]. This is not a more probable alternative explanation in cases of immediate stroke after CSM.

In cases of non-immediate stroke, where stroke follows CSM within hours, days, or weeks, it is more likely than not that dissection progressed into stroke independent of the application of CSM, as there is no mechanism of causation of a non-immediate stroke by CSM. However, that does not mean that progression was inevitable. Most dissections of the vertebral arteries heal spontaneously and especially extracranial VADs generally carry a good prognosis [54].

Murphy acknowledges that the onset of a stroke may be “hastened” by CSM but presents no mechanism for how CSM could “hasten” the onset of stroke. This study has presented mechanisms that explain how CSM could cause immediate stroke.

Far from being inevitable, with proper diagnosis and treatment, dissection rarely progresses into stroke. In general, individuals with VAD appear to have relatively good outcomes when treated in a routine clinical fashion [40]. When CAD is diagnosed and referred for emergency medical care, the chance of avoiding stroke is almost 100% [55].

Suggestions for further research

In a patient susceptible to CAD [25], it seems plausible that CSM could cause CAD, and the intimal flap could obstruct the cervical artery or a branch vessel, impede blood flow to the brain, and cause immediate post-manipulative ischemic stroke. However, no study was found that proposed an intimal flap mechanism of causation of stroke from CSM in a cervical artery. Research into this mechanism of causation is indicated.

Clinical practice guidelines to inform the standard of care in this area are needed for physicians and medicolegal professionals. Such guidelines should be based on thromboembolic and thrombotic mechanisms of causation and the clinical risk assessment strategies from Chaibi and Russell [38] and Rushton et al. [50].

Epidemiological studies with an immediate cohort and temporality established by the medical records are needed. In Whedon et al.’s 2015 cohort study [26] of the risk of stroke after CSM, 55 cases of stroke that occurred on the same day as a DC office visit were excluded. In the 0-1-day cohort, Whedon et al. did not have the data to determine if the stroke occurred after the DC office visit; therefore, the authors *speculated* that the stroke occurred before the DC visit. Meaningful conclusions on the risk of immediate stroke after CSM can only be reached if medical records are reviewed to determine temporality.

Limitations

This is a narrative review, rather than a systematic review. As article screening and data extraction were done by a single author, it is possible that relevant articles may have been missed, or that there may have been errors in extraction.

Another limitation is that only two literature databases were searched. Future research could be improved by searching databases from physiotherapy, osteopathic, naturopathic, neurology, and emergency medicine professions. Other databases that could also be searched include EBSCOhost, Scopus, Web of Science, and Google Scholar.

## Conclusions

There are plausible thromboembolic and thrombotic mechanisms of causation of immediate stroke by CSM in the literature. The common premise of these mechanisms is CAD being present before CSM, and not occurring as a result of CSM. These mechanisms of causation have clinical and medicolegal implications for physicians performing CSM.

## References

[1] Biller J, Sacco RL, Albuquerque FC (2014). Cervical arterial dissections and association with cervical manipulative therapy: a statement for healthcare professionals from the american heart association/american stroke association. Stroke.

[2] Cassidy JD, Boyle E, Côté P, He Y, Hogg-Johnson S, Silver FL, Bondy SJ (2008). Risk of vertebrobasilar stroke and chiropractic care: results of a population-based case-control and case-crossover study. Spine (Phila Pa 1976).

[3] Terrett AG (2001). Current Concepts in Vertebrobasilar Complications Following Spinal Manipulation. West Des Moines, Iowa: NCMIC.

[4] Cassidy JD, Boyle E, Côté P, Hogg-Johnson S, Bondy SJ, Haldeman S (2017). Risk of carotid stroke after chiropractic care: a population-based case-crossover study. J Stroke Cerebrovasc Dis.

[5] Symons B, Herzog W (2013). Cervical artery dissection: a biomechanical perspective. J Can Chiropr Assoc.

[6] Church EW, Sieg EP, Zalatimo O, Hussain NS, Glantz M, Harbaugh RE (2016). Systematic review and meta-analysis of chiropractic care and cervical artery dissection: no evidence for causation. Cureus.

[7] Haldeman S, Carey P, Townsend M, Papadopoulos C (2001). Arterial dissections following cervical manipulation: the chiropractic experience. CMAJ.

[8] L. Smith, “25 “25 (2024). 25 chiropractic statistics (and facts): crunching the numbers. Good Body.

[9] Chu EC, Trager RJ, Lee LY, Niazi IK (2023). A retrospective analysis of the incidence of severe adverse events among recipients of chiropractic spinal manipulative therapy. Sci Rep.

[10] Rothwell DM, Bondy SJ, Williams JI (2001). Chiropractic manipulation and stroke: a population-based case-control study. Stroke.

[11] Kosloff TM, Elton D, Tao J, Bannister WM (2015). Chiropractic care and the risk of vertebrobasilar stroke: results of a case-control study in U.S. commercial and Medicare Advantage populations. Chiropr Man Therap.

[12] Kennell KA, Daghfal MM, Patel SG, DeSanto JR, Waterman GS, Bertino RE (2017). Cervical artery dissection related to chiropractic manipulation: one institution's experience. J Fam Pract.

[13] Whedon JM, Petersen CL, Li Z, Schoelkopf WJ, Haldeman S, MacKenzie TA, Lurie JD (2022). Association between cervical artery dissection and spinal manipulative therapy -a medicare claims analysis. BMC Geriatr.

[14] Whedon JM, Petersen CL, Schoellkopf WJ, Haldeman S, MacKenzie TA, Lurie JD (2023). The association between cervical artery dissection and spinal manipulation among US adults. Eur Spine J.

[15] Norris JW, Beletsky V, Nadareishvili ZG (2000). Sudden neck movement and cervical artery dissection. The Canadian Stroke Consortium. CMAJ.

[16] Mann T, Refshauge KM (2001). Causes of complications from cervical spine manipulation. Aust J Physiother.

[17] Moser N, Mior S, Noseworthy M, Côté P, Wells G, Behr M, Triano J (2019). Effect of cervical manipulation on vertebral artery and cerebral haemodynamics in patients with chronic neck pain: a crossover randomised controlled trial. BMJ Open.

[18] Achalandabaso A, Plaza-Manzano G, Lomas-Vega R (2014). Tissue damage markers after a spinal manipulation in healthy subjects: a preliminary report of a randomized controlled trial. Dis Markers.

[19] Mas JL, Henin D, Bousser MG, Chain F, Hauw JJ (1989). Dissecting aneurysm of the vertebral artery and cervical manipulation: a case report with autopsy. Neurology.

[20] Haldeman S, Kohlbeck FJ, McGregor M (1999). Risk factors and precipitating neck movements causing vertebrobasilar artery dissection after cervical trauma and spinal manipulation. Spine (Phila Pa 1976).

[21] Haldeman S, Kohlbeck FJ, McGregor M (2002). Stroke, cerebral artery dissection, and cervical spine manipulation therapy. J Neurol.

[22] Smith WS, Johnston SC, Skalabrin EJ, Weaver M, Azari P, Albers GW, Gress DR (2003). Spinal manipulative therapy is an independent risk factor for vertebral artery dissection. Neurology.

[23] Schwartz NE, Vertinsky AT, Hirsch KG, Albers GW (2009). Clinical and radiographic natural history of cervical artery dissections. J Stroke Cerebrovasc Dis.

[24] Albuquerque FC, Hu YC, Dashti SR (2011). Craniocervical arterial dissections as sequelae of chiropractic manipulation: patterns of injury and management. J Neurosurg.

[25] Tuchin P (2013). Chiropractic and stroke: association or causation?. Int J Clin Pract.

[26] Whedon JM, Song Y, Mackenzie TA, Phillips RB, Lukovits TG, Lurie JD (2015). Risk of stroke after chiropractic spinal manipulation in medicare B beneficiaries aged 66 to 99 years with neck pain. J Manipulative Physiol Ther.

[27] Thomas LC (2016). Cervical arterial dissection: an overview and implications for manipulative therapy practice. Man Ther.

[28] Neeb L, Reuter U (2016). Stroke after chiropractic manipulations. Treatment-Related Stroke.

[29] Murphy DR (2010). Current understanding of the relationship between cervical manipulation and stroke: what does it mean for the chiropractic profession?. Chiropr Osteopat.

[30] Johnson CP, Lawler W, Burns J (1993). Use of histomorphometry in the assessment of fatal vertebral artery dissection. J Clin Pathol.

[31] Hill AB (1965). The environment and disease: association or causation?. Proc R Soc Med.

[32] Wynd S, Anderson T, Kawchuk G (2008). Effect of cervical spine manipulation on a pre-existing vascular lesion within the canine vertebral artery. Cerebrovasc Dis.

[33] Demetrious JS (2018). Spontaneous cervical artery dissection: a fluoroquinolone induced connective tissue disorder?. Chiropr Man Therap.

[34] (2024). Labpedia.net. Clotting time. https://labpedia.net/clotting-time-c-t/.

[35] Hufnagel A, Hammers A, Schönle PW, Böhm KD, Leonhardt G (1999). Stroke following chiropractic manipulation of the cervical spine. J Neurol.

[36] (2024). Association of Chiropractic Colleges Informed Consent Guideline. https://www.chirocolleges.org.

[37] Lehman JJ, Conwell TD, Sherman PR (2008). Should the chiropractic profession embrace the doctrine of informed consent?. J Chiropr Med.

[38] Chaibi A, Russell MB (2019). A risk-benefit assessment strategy to exclude cervical artery dissection in spinal manual-therapy: a comprehensive review. Ann Med.

[39] Hutting N, Kranenburg HA, Kerry R (2020). Yes, we should abandon pre-treatment positional testing of the cervical spine. Musculoskelet Sci Pract.

[40] Gottesman RF, Sharma P, Robinson KA, Arnan M, Tsui M, Ladha K, Newman-Toker DE (2012). Clinical characteristics of symptomatic vertebral artery dissection: a systematic review. Neurologist.

[41] Thanvi B, Munshi SK, Dawson SL, Robinson TG (2005). Carotid and vertebral artery dissection syndromes. Postgrad Med J.

[42] Kropp P, Egli G, Sándor PS (Kropp P). Tension-type headache: introduction and diagnostic criteria. Handbook of Clinical Neurology.

[43] (2018). Headache Classification Committee of the International Headache Society (IHS) The International Classification of Headache Disorders, 3rd edition. Cephalalgia.

[44] Mattox R, Smith LW, Kettner NW (2014). Recognition of spontaneous vertebral artery dissection preempting spinal manipulative therapy: a patient presenting with neck pain and headache for chiropractic care. J Chiropr Med.

[45] Easton JD, Johnston SC (2022). Time to retire the concept of transient ischemic attack. JAMA.

[46] Arnold M, Cumurciuc R, Stapf C, Favrole P, Berthet K, Bousser MG (2006). Pain as the only symptom of cervical artery dissection. J Neurol Neurosurg Psychiatry.

[47] Tuchin P (2012). A replication of the study 'Adverse effects of spinal manipulation: a systematic review'. Chiropr Man Therap.

[48] Lee VH, Brown RD Jr, Mandrekar JN, Mokri B (2006). Incidence and outcome of cervical artery dissection: a population-based study. Neurology.

[49] Haldeman S, Kohlbeck FJ, McGregor M (2002). Unpredictability of cerebrovascular ischemia associated with cervical spine manipulation therapy: a review of sixty-four cases after cervical spine manipulation. Spine (Phila Pa 1976).

[50] Rushton A, Carlesso LC, Flynn T, Hing WA, Rubinstein SM, Vogel S, Kerry R (2023). International framework for examination of the cervical region for potential of vascular pathologies of the neck prior to musculoskeletal intervention: International IFOMPT Cervical Framework. J Orthop Sports Phys Ther.

[51] Ahuja AS (2019). Should RCT's be used as the gold standard for evidence based medicine?. Integr Med Res.

[52] Freeman MD (2018). A practicable and systematic approach to medicolegal causation. Orthopedics.

[53] Gorrell LM, Sawatsky A, Edwards WB, Herzog W (2023). Vertebral arteries do not experience tensile force during manual cervical spine manipulation applied to human cadavers. J Man Manip Ther.

[54] Park KW, Park JS, Hwang SC, Im SB, Shin WH, Kim BT (2008). Vertebral artery dissection: natural history, clinical features and therapeutic considerations. J Korean Neurosurg Soc.

[55] Morris NA, Merkler AE, Gialdini G, Kamel H (2017). Timing of incident stroke risk after cervical artery dissection presenting without ischemia. Stroke.

